# Population perception of mandatory childhood vaccination programme before its implementation, France, 2017

**DOI:** 10.2807/1560-7917.ES.2019.24.25.1900053

**Published:** 2019-06-20

**Authors:** Pauline Mathieu, Arnaud Gautier, Jocelyn Raude, Thomas Goronflot, Titouan Launay, Marion Debin, Caroline Guerrisi, Clément Turbelin, Thomas Hanslik, Christine Jestin, Vittoria Colizza, Thierry Blanchon, Louise Rossignol

**Affiliations:** 1 Sorbonne Universités, INSERM, Institut Pierre Louis d’épidémiologie et de Santé Publique (IPLESP), F-75012, Paris, France; 2 Santé Publique France, Saint-Maurice, France; 3 EHESP Rennes, Université Sorbonne Paris Cité, France; 4 Université de Versailles Saint-Quentin-en-Yvelines, UVSQ, UFR de Médecine, FR-78000, Versailles, France; 5 Service de Médecine Interne, Hôpital Ambroise Paré, Assistance Publique - Hôpitaux de Paris, APHP, FR-92100, Boulogne Billancourt, France

**Keywords:** immunisation, mandatory vaccination, vaccine hesitancy, France, vaccine-preventable diseases, public health policy, vaccines, immunisation

## Abstract

**Background:**

Vaccination policy in France was previously characterised by the coexistence of eight recommended and three mandatory vaccinations for children younger than 2 years old. These 11 vaccines are now mandatory for all children born after 1 January 2018.

**Aim:**

To study the French population’s opinion about this new policy and to assess factors associated with a positive opinion during this changing phase.

**Methods:**

A cross-sectional survey about vaccination was conducted from 16 November–19 December 2017 among the GrippeNet.fr cohort. Data were weighted for age, sex and education according to the French population. Univariate and multivariate analyses were performed to identify factors associated with a favourable opinion on mandatory vaccines’ extension and defined in the ‘3Cs’ model by the World Health Organization Strategic Advisory Group of Experts working group on vaccine hesitancy.

**Results:**

Of the 3,222 participants (response rate 50.5%) and after adjustment, 64.5% agreed with the extension of mandatory vaccines. It was considered a necessary step by 68.7% of the study population, while 33.8% considered it unsafe for children and 56.9% saw it as authoritarian. Factors associated with a positive opinion about the extension of mandatory vaccines were components of the confidence, complacency and convenience dimensions of the ‘3Cs’ model.

**Conclusions:**

In our sample, two thirds of the French population was in favour of the extension of mandatory vaccines for children. Perception of vaccine safety and benefits were major predictors for positive and negative opinions about this new policy.

## Introduction

Vaccination suffers in several countries from growing scepticism [1,2]. This complex phenomenon, also known as ‘vaccine hesitancy’, is defined by the World Health Organization (WHO) as a ‘delay in acceptance or refusal of vaccines despite availability of vaccination services’ [3]. According to the Strategic Advisory Group of Experts (SAGE) working group on vaccine hesitancy, vaccination determinants belong to the ‘3Cs’ model, composed of confidence, convenience and complacency factors [4]. The confidence dimension refers to the trust in the effectiveness and safety of vaccines, in the system that delivers them and in the motivations of vaccination policymakers. The complacency dimension refers to the perception that vaccination is still a necessary preventive action and the convenience dimension refers to availability and accessibility of vaccines [4]. In France, the confidence dimension has been weakened by several controversies; for example, by claims that the hepatitis B virus (HBV) vaccine might be linked to multiple sclerosis or by safety concerns about human papillomavirus (HPV) vaccine, even though no scientific data support these theories [5,6]. The mass vaccination campaign to protect the French population against the pandemic influenza A(H1N1) in 2009 also appears to have affected population confidence in vaccine safety. The accelerated authorisation procedure to market pandemic vaccines called their efficacy and safety into question, as well as the actual motivations of pharmaceutical firms. Moreover, the public health authorities lost credibility because of the contrast between the large size of the vaccination campaign and the small proportion of the population that was actually vaccinated during the pandemic [7]. Information sources used by the general population may also influence beliefs about vaccine safety and efficacy, attitude towards vaccination and the level of knowledge about vaccines [8-10]. Several studies have shown that health professionals’ recommendations have a positive influence on vaccination behaviour, whereas the Internet has played a large role in disseminating anti-vaccination information [8,9,11]. Negative content related to vaccination tends to proliferate on the Internet, where anti-vaccination arguments are more present, have greater visibility and are rarely countered [10].

In order to address vaccine hesitancy and thus improve vaccination coverage, several new measures were set up in France. For example, in 2016 the national public health agency (Santé publique France) launched the website *Vaccination Info Service* to provide reliable information about vaccination [12]. Concerning the influenza vaccine, since 2017 French government have allowed pharmacists to administer vaccines to adults who have already had a vaccination in the past in order to increase convenience and expand access to vaccination. A new vaccination policy for children was also set up in France in 2018.

Until 2018, French vaccination policy was characterised by the coexistence of recommended and mandatory vaccinations. For newborns, measles-mumps-rubella (MMR), pertussis, pneumococcus, HBV, meningitis C and *Haemophilus influenzae* vaccinations were recommended, whereas diphtheria, tetanus and poliomyelitis (DT-polio) vaccinations were mandatory.

In 2004, the French public health law set a vaccination coverage goal of 95% for children vaccine-preventable diseases. In 2015, only one childhood vaccine reached and surpassed that goal: the mandatory DT-polio vaccine, with 99% coverage. Coverage for three doses of HBV vaccine was estimated at 88%, for two doses of MMR vaccine at 80% and for at least one dose of meningococcal vaccine at 78% by the age of 24 months [13]. A French study revealed that non-mandatory vaccinations were perceived as optional and not as safe and effective as mandatory ones [14]. In order to raise vaccination coverage and restore trust in vaccines, the French government decided to make all eight recommended vaccines mandatory for all children born after 1 January, 2018 [13,15,16]. Public opinion was central to this decision. Indeed, this measure resulted from a citizen consultation on vaccination that took place in 2016, in which the point of view of various groups was analysed: the general population, health professionals, researchers in the humanities and social sciences, and experts on vaccines [16]. However, some studies showed that policies with mandatory vaccination have been controversial, especially in a context of mistrust towards vaccination [17], and could generate opposition from anti-vaccine activists [16,18].

Vaccination policies vary widely between European countries, from no recommended vaccines at all, to entirely mandatory childhood vaccination programmes [19]. In Italy, the low immunisation levels and negative trends also led to the introduction of mandatory vaccination in July 2017 for 10 infectious diseases [11,19]. A few months before this new obligation, an Italian study found that the majority of 1,820 interviewed pregnant women (81.6%) were in favour of compulsory vaccination and that information sources and confidence towards health professionals were the main determinants of acceptance of mandatory vaccines [11].

The main objective of this study is to assess the French population’s acceptance of this new mandatory vaccine policy in France and to identify factors associated with its favourable regard during this transitional phase in the end of 2017, in order to guide future public health policies.

## Methods

We conducted a cross-sectional survey on GrippeNet.fr participants from November–December 2017, just before implementation of the new vaccination policy in France.

### Population

The study was conducted using data collected in the cohort GrippeNet.fr, a web-based participative study conducted in France since 2012 [20]. This project is part of a European multicentric project, Influenzanet (http://www.influenzanet.eu), which allows monitoring of influenza-like illness diffusion directly in the general population. The inclusion criteria to participate in the GrippeNet.fr study include: residence in France and access to the Internet. Upon registration, participants are asked to complete a baseline questionnaire covering demographic factors (age, sex), geographical factors (location of home and work/school, expressed at the municipality level), socio-economic factors (household size and composition, occupation, educational level, number of daily contacts with children or elderly people, daily transportation means) and several health-related factors. Subsequently, they are invited to describe weekly clinical symptoms during the influenza season. According to a previous study, the GrippeNet.fr population was not representative of the general population in terms of age and sex; however, all age groups were represented, including older age groups (≥ 65 years old). Once adjusted for age and sex, the GrippeNet.fr population was found to be more frequently employed, with a higher education level and vaccination rate than the general population (data from 2012 [20]).

For this study, participants in GrippeNet.fr were encouraged from 16 November–19 December 2017 to complete a questionnaire on the theme of vaccination, in addition to the weekly symptom survey. At that time, the new mandatory vaccination policy was approved by the government and was planned to start for all children born after 1 January 2018. An email and a reminder were sent to invite GrippeNet.fr participants to take part in this study. Participation was voluntary.

### Inclusion criteria

From the GrippeNet.fr participant pool, we included only participants who: were between 18–90 years old, completed at least one baseline questionnaire, were living in mainland France, had participated in 2016/17 or 2017/18 GrippeNet.fr seasons by filling in at least one questionnaire on weekly clinical symptoms.

### Sample size calculation

A previous study showed that around 56% of the French population was in favour of the extension of the mandatory vaccination in 2008 [21]. Considering this proportion, we set a confidence level at 95% and 5% margin of error. The final sample size was expected to be at least 1,208 completed questionnaires.

#### Questionnaire

The questionnaire was built according to the literature [22,23]. It was then discussed and validated by a panel of experts in the vaccination field: members of the national public health agency (Santé publique France), immunologists, epidemiologists, a general practitioner and a sociologist, with support from biostatisticians. The survey included 36 questions, either optional or mandatory, about vaccination. Five of them were multiple-answers questions, 19 were single-choice questions, seven were numerical scale from 0 to 100 questions and five were free text questions (not analysed here).

Questions were divided into three main categories: (i) behaviour, awareness and opinion towards vaccination (influenza vaccination in the current season, feeling well-informed about vaccines, sources of information towards vaccination, trust in different sources), (ii) perceived risks and benefits of vaccination (population health benefits, individual health benefits, inconveniences, side effects, vaccine testing) and (iii) opinion on the extension to 11 mandatory vaccines.

Socio-demographic characteristics came from baseline questionnaires: age, sex, level of education, occupation, presence of children in the household, place of residence and geographic division (according to French phone area codes).

Questions on vaccine benefits and risk perception were evaluated with a numeric scale ranging from 0 to 100, where 0 meant least benefits, inconvenience, probability and seriousness and 100 meant most benefits, inconvenience, probability and seriousness. Inconvenience of vaccination meant both logistical and physical inconvenience of vaccination (time, money, puncture pain, etc.).

#### Data analyses

A description of the study population was performed and outliers were verified, corrected or excluded as needed. Duplicate questionnaires were removed (the last questionnaire completed was kept for analyses).

The French National Institute of Statistics and Economic Studies (INSEE) provided the demographic and socio-economic data of the French population.

Some variables were recoded in order to facilitate the analyses and the presentation of the results. The place of residence was defined in two categories (urban or rural), based on the geographical location and according to the INSEE definition. The opinion about new mandatory vaccines and several others variables, were split into two levels, ‘in favour’ (grouping ‘strongly agree’ and ‘agree’ together) and ‘not in favour’ (grouping ‘neither agree nor disagree’, ‘disagree’ and ‘strongly disagree’ together). We classified the neutral answer (‘neither agree nor disagree’) within the negative opinion for analyses, as neutrality may reveal either a lack of perceived benefits or doubts over the successful implementation of mandatory vaccinations. Several authors of studies on vaccine hesitancy have adopted a similar approach [1,7]. The quantitative variable concerning level of trust in institutional sources was split in two levels, ‘in favour’ for a score > 50 of 100 and ‘not in favour’ for a score ≤ 50 of 100. Other quantitative variables were stratified into quartiles, except for age, for which age groups were created: 18–34 years old, 35–64 years old, 65–90 years old.

Survey respondents were weighted to reflect the French population’s proportions on age, sex and level of education, based on the most recent INSEE data available [20]. For descriptive analysis, we expressed the raw number, the raw and weighted proportions of the qualitative variables, and the weighted median and quartiles of the quantitative variables.

To assess the factors associated with positive opinions about the new mandatory vaccines, weighted populations were used in regression models. The effect of each explanatory variable was studied using univariate analysis first, then multivariate analysis. All collected variables were assessed by univariate analysis, and those achieving a p value < 0.20 (using the Wald test for logistic regression) and considered relevant by the authors were included in multivariate analysis. We used a principal component analysis (PCA) to identify independent dimensions of patient trust in sources of information to limit factors included in the multivariate analysis. Sources of information that contributed to the same dimension in PCA were grouped in a unique variable. A backward stepwise variable selection procedure was then used to remove factors with a p value > 0.05. Adjusted odds ratios (aORs) and 95% CIs were calculated for the determinants that remained in the final model. Missing values were indicated and were excluded from the models. All statistical analyses were performed using the R software version 3.5.0 (R Foundation, Vienna, Austria).

### Ethical statement

This study was conducted in agreement with French regulations on privacy and data collection and treatment and was approved by the Comité Consultatif sur le Traitement de l’Information en matière de Recherche (CCTIRS, Advisory committee on information processing for research, authorisation 11.565) and by the Commission Nationale de l’Informatique et des Libertés (CNIL, French Data Protection Authority, authorisation DR-2012–024).

## Results

Among the 6,383 GrippeNet.fr participants who fulfilled inclusion criteria, 3,222 individuals participated. The response rate was 50.5% (3,222/6,383). Duplicate questionnaires were removed (n = 63).

### Socio-demographic characteristics

Before adjustment, the study population was composed of 62.9% women and 37.1% men, with a mean age of 52.7 years; 66.3% of respondents had a level of education higher than high school diploma.

After adjustment for age, sex and level of education, data showed that a majority of the population was working (51.4%) and 38.9% was retired. Most of the population was living in urban areas (76.8%) and with children (76.2%). Influenza vaccination coverage for people ≥ 65 years old was 60.9%. All the following results are adjusted (Table 1).

**Table 1:** Socio-demographic characteristics of survey respondents, perception of mandatory childhood vaccination programme study, France, 2017 (n = 3,222)

Socio-demographic characteristics	Data from GrippeNet survey			French population data (%)
	Raw number	Raw percentage (%)	Weighted percentage^a^ (%)	
**Sex**				
Female	2,027	62.9	52.4	52.4
Male	1,195	37.1	47.6	47.6
**Age (years)**				
18–34	256	7.9	20.9	20.9
35–64	1,807	56.1	54.0	54.0
65–90	1,159	36.0	25.1	25.1
**Level of education**				
High school diploma	605	18.8	16.7	16.7
> High school diploma	2,135	66.3	27.8	27.8
< High school diploma	482	14.7	55.6	55.6
**Occupation**				
Working	1,551	48.8	51.4	53
Student	26	0.8	2.3	4
Unemployed	67	2.1	2.7	5
Stay at home/sick leave	128	4.0	4.7	38^b^
Retired	1,409	44.3	38.9	
**Household composition**				
Living with children	2,436	75.8	76.2	NA
Living without children	778	24.2	23.8	NA
**Place of residence**				
Rural	609	18.9	23.2	25
Urban	2,613	81.1	76.8	75
**Geographic division (according to French phone area codes)**				
1 – Île-de-France (including Paris)	933	28.9	15.7	19
2 – North West	656	20.4	19.5	20
3 – North East	317	19.1	21.1	22
4 – South West	564	17.5	30.3	25
5 – South East	452	14.0	13.4	14

NA: data not available.

^a^ Weighted on age, sex and level of education of the French population data.

^b^ Percentage of ‘Stay at home/sick leave’ and ‘Retired’ in the overall French population

### Behaviour, awareness and opinion towards vaccination

**Figure 1:**
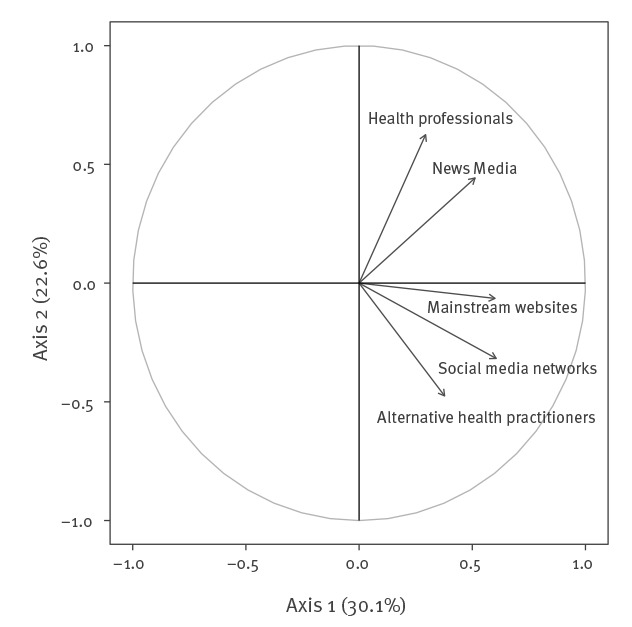
Results of the principal component analysis on the level of trust in different sources of vaccination information, perception of mandatory childhood vaccination programme study, France, 2017

A large majority of respondents had only a paper vaccination record (76.1%) and thought of themselves as being up to date with the immunisation schedule (80.4%). Santé publique France’s website about vaccination was little known by the study population (11.6%), whereas individuals gathered information about vaccination mostly from health professionals (68.1%), news media (49.2%) and institutional sources (45.0%). Trust in health professionals was very high (85.4%), whereas less than half of the study population trusted information delivered by the news media (47.9%) and very few trusted information found on social media networks (7.7%). Principal component analysis resulted in identifying two independent dimensions of trust in sources of information: on the one hand, a dimension of respondents who trusted health professionals or news media and on the other hand, a dimension of respondents who trusted social networks, mainstream websites or alternative health practitioners (Figure 1).

Concerning the administration of vaccination, 67.5% and 62.7% of the population approved vaccination by pharmacists and occupational physicians, respectively (Table 2 **,** Figure 1).

**Table 2:** Survey respondents’ behaviour towards vaccination, perception of mandatory childhood vaccination programme study, France, 2017 (n = 3,222)

Survey responses	Raw number	Raw percentage (%)	Weighted percentage^a^ (%)
**Influenza vaccination in the current season**			
Yes	1,494	46.4	38.9
No	1,728	53.6	61.1
**Has a vaccination record**			
Paper format	2,403	74.6	76.1
Electronic format	40	1.4	0.7
Both	60	1.9	2.0
None	647	20.1	19.1
Don’t know	72	2.2	2.1
**Declares to be up to date with immunisation schedule**			
Yes	2,603	80.9	80.4
No	486	15.1	13.7
Don’t know	133	4.1	5.9
**Aware of Santé publique France’s website about vaccination^b^**			
Yes	377	11.7	11.6
No	2,845	86.4	86.3
**Feels well informed about vaccination**			
Yes	2,495	77.4	73.7
No	727	22.6	26.3
**Consults as a vaccination information source (multiple-answers question)**			
News media	1,547	48.0	49.2
Health professionals	2,181	67.7	68.1
Institutional sources	1,559	48.4	45.0
Scientific publications	672	20.9	16.7
Mainstream websites	443	13.7	14.9
Alternative health practitioners	261	8.1	9.4
Social media networks	130	4.0	6.2
None/Don’t know	151	4.7	5.0
**Trusts as an information source (multiple-answers question)**			
News media	1,540	47.8	47.9
Health professionals	2,762	85.7	85.4
Mainstream websites	1,067	33.1	36.9
Alternative medicine professionals	1,255	39.0	45.5
Social media networks	138	4.3	7.7
**Trusts as an information source (grouping by principal component analysis)**			
Practitioners and/or news media (missing values: 235)	2,784	93.2	92.0
Social media network and/or mainstream websites and/or alternative practitioners (missing values: 303)	1,110	38.0	42.1
**Personal reasons for getting vaccinated (multiple-answers question)**			
Obligation only	184	5.7	9.0
Individual protection	2,726	84.6	80.2
Family protection	2,167	67.3	61.1
Population protection	2,262	70.2	59.6
None/Don’t know	94	2.9	4.3
**Thinks vaccines are thoroughly tested**			
Yes	2,434	75.5	70.4
No	788	24,5	29,6
**In favour of vaccination by pharmacists**			
Yes	2,356	73.1	67.5
No/Don’t know	866	26.9	32.5
**In favour of vaccination by occupational physician (missing values: 50)**			
Yes	2,108	66.4	62.7
No	297	9.4	14.4
Not concerned	817	24.2	22.8

^a^ Weighted on age, sex and level of education of the French population.

^b^ In 2016, the national public health agency (Santé publique France) launched the website *Vaccination Info Service* to provide reliable information about vaccination to the general population in France.

### Perception of vaccination benefits and risks

**Figure 2:**
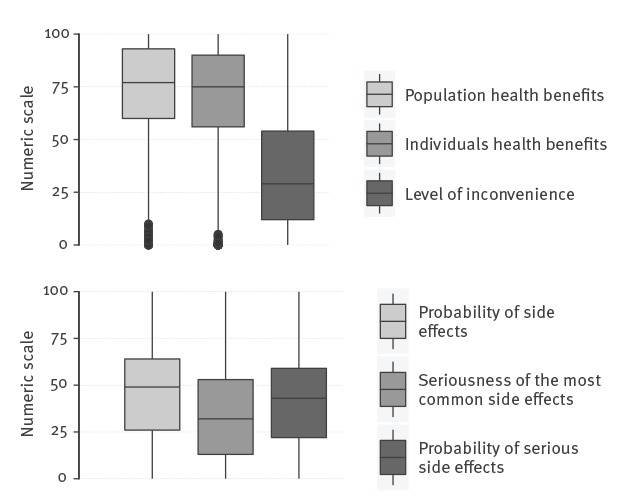
Benefits and risks of vaccination, perception of mandatory childhood vaccination programme study, France, 2017 (n = 3,222)

Evaluation of the benefits of vaccination on individual and population health on a scale of 0 to 100, had a median score of 75.0 (interquartile range (IQR): 56.0–89.7) and 77.0 (IQR: 60.0–93.0), respectively. The median level of inconvenience was estimated at 29.0 (IQR: 12.0–54.0). The probability of side effects of any type and of serious side effects had a median of 49.0 (IQR: 26.0–64.0) and 32.0 (IQR: 13.0–53.0), respectively. Evaluation of the seriousness of the most common side effects (without specifying these side effects) had a median of 43.0 (IQR: 22.0–59.0) (Figure 2).

Perceptions were scored on a scale of 0 to 100, with 0 meaning the lowest benefit, level of inconvenience, probability or seriousness and 100 meaning the highest benefit, level of inconvenience, probability or seriousness, respectively.

### Opinions on vaccination and the extension to 11 mandatory vaccines

A large majority of the respondents supported vaccination in general (81.7%); however, 28.2% were not in favour of some specific vaccines. Concerning the new mandatory vaccination policy, 62.4% were in favour and 31.0% were not in favour (including 6.5% with no opinion). The new programme was considered to be a necessary step for 68.7% of the population, whereas 33.8% of participants regarded it to be a risk for children who will be vaccinated. The policy change was perceived as authoritarian by 56.9% of respondents (Table 3).

**Table 3:** Survey respondents’ opinion of new mandatory vaccines in France, perception of mandatory childhood vaccination programme study, France, 2017 (n = 3,222)

Survey responses	Raw number	Raw percentage (%)	Weighted percentage^a^ (%)
**In favour of vaccination in general (missing values 10)**			
Strongly agree	1,746	54.2	46.8
Agree	1,098	34.1	34.9
Disagree	288	8.9	13.8
Strongly disagree	80	2.5	4.3
**Not in favour of some specific vaccines (missing values: 44)**			
Yes	896	28.2	27.8
No	2,287	71.8	71.0
**In favour of extension to 11 mandatory vaccines**			
Strongly agree	1,123	34.9	29.6
Agree	1,011	31.4	32.8
Disagree	474	14.7	14.3
Strongly disagree	446	13.8	16.7
Neither agree nor disagree	168	5.2	6.5
**New mandatory vaccines are as important as those already mandatory**			
Strongly agree	977	30.3	26.4
Agree	1,133	35.2	37.6
Disagree	561	17.4	17.5
Strongly disagree	286	8.9	10.4
Neither agree nor disagree	265	8.2	8.2
**This is a necessary step (missing values:249*)***			
Strongly agree	1,276	42.9	36.6
Agree	974	32.8	32.1
Disagree	406	13.7	10.5
Strongly disagree	215	7.2	9.7
Neither agree nor disagree	102	3.4	3.3
**This measure is putting children who will be vaccinated at risk (missing values: 307)**			
Strongly agree	332	11.4	11.7
Agree	555	19.0	22.1
Disagree	1,185	40.7	31.8
Strongly disagree	594	20.4	15.0
Neither agree nor disagree	249	8.5	10.2
**This is an authoritarian measure (missing values 322)**			
Strongly agree	931	32.1	28.6
Agree	930	32.1	28.3
Disagree	563	19.4	18.6
Strongly disagree	338	11.7	9.9
Neither agree nor disagree	138	4.8	5.0

^a^ Weighted on age, sex and level of education of the French population.

### Factors associated with a favourable opinion of the extension to 11 mandatory vaccines

In univariate analysis, factors associated with a favourable attitude towards the extension to 11 mandatory vaccines were both socio-demographic and concerning behaviour and opinions towards vaccination (**Table 4**).

**Table 4:** Univariate analysis for predicting favourable attitudes towards new mandatory vaccines, perception of mandatory childhood vaccination programme study, France, 2017 (n = 3,222)

Survey responses	OR (95% CI)	p value^a^
**Sex**		
Female	Ref.	0.03
Male	1.40 (1.03–1.91)	
**Age (years)**		
35–64	Ref.	0.33
18–34	1.16 (0.66–2.02)	
65–90	0.84 (0.65–1.09)	
**Level of education**		
High school diploma	Ref.	< 10 ^− 4^
> High school diploma	1.77 (1.25–2.51)	
< High school diploma	1.02 (0.68–1.52)	
**Occupation**		
Working	Ref.	0.20
Student	2.22 (0.74–6.68)	
Unemployed	0.53 (0.24–1.18)	
Stay at home/sick leave	0.72 (0.40–1.28)	
Retired	0.88 (0.65–1.19)	
**Household composition**		
Living without children	Ref.	0.94
Living with children	0.99 (0.67–1.45)	
**Influenza vaccination in the current season**		
No/Don’t know	Ref.	
Yes	2.75 (1.98–3.80)	< 10 ^− 4^
**Place of residence**		
Rural	Ref.	0.004
Urban	1.69 (1.19–2.42)	
**Feels well informed about vaccines**		
No	Ref.	< 10 ^− 4^
Yes	2.24 (1.63–3.06)	
**Trusts as a vaccination information source**		
News media	2.46 (1.81–3.33)	< 10 ^− 4^
Health professionals	18.99 (10.10–35.70)	< 10 ^− 4^
Institutional sources	10.63 (7.77–14.56)	< 10 ^− 4^
Mainstream websites	1.19 (0.85–1.68)	0.31
Alternative health practitioners	0.51 (0.36–0.71)	< 10 ^− 4^
Social media networks	0.49 (0.24–0.98)	0.04
**Trusts as a vaccination information source (grouped by principal component analysis)**		
Health professionals and/or news media (missing values: 235)	18.52 (10.01–34.25)	< 10 ^− 4^
Social media networks and/or mainstream websites and/or alternative health practitioners (missing values: 303)	1.16 (0.83–1.61)	0.37
**Perceived population health benefits**		
Q1 (least benefits)	Ref.	< 10 ^− 4^
Q2	4.07 (2.73–6.07)	
Q3	12.71 (8.46–19.10)	
Q4 (most benefits)	36.60 (22.27–60.15)	
**Perceived individual health benefits**		
Q1 (least benefits)	Ref.	< 10 ^− 4^
Q2	5.08 (3.58–7.22)	
Q3	10.61 (6.92–16.27)	
Q4 (most benefits)	19.90 (12.76–31.03)	
**Perceived level of inconvenience**		
Q4 (most inconvenient)	Ref.	< 10 ^− 4^
Q3	1.21 (0.81–1.82)	
Q2	2.67 (1.72–4.15)	
Q1 (least inconvenient)	4.60 (3.00–7.06)	
**Perceived probability of side effects**		
Q4 (most probable)	Ref.	< 10 ^− 4^
Q3	3.40 (2.27–5.09)	
Q2	5.38 (3.42–8.46)	
Q1 (least probable)	16.70 (10.11–27.60)	
**Perceived seriousness of the most common side effects**		
Q4 (most serious)	Ref.	< 10 ^− 4^
Q3	2.52 (1.66–3.82)	
Q2	6.40 (4.35–9.43)	
Q1 (least serious)	17.67 (11.53–27.09)	
**Perceived probability of serious side effect**		
Q4 (most probable)	Ref.	< 10 ^− 4^
Q3	1.68 (1.15–2.46)	
Q2	4.36 (2.64–7.21)	
Q1 (least probable)	13.09 (8.14–21.03)	
**Personal reasons for getting vaccinated**		
Protection (personal, family, population)	Ref.	< 10 ^− 4^
Obligation only	0.13 (0.05–0.33)	
None/Don’t know	0.14 (0.06–0.31)	
**Thinks vaccines are thoroughly tested**		
No	Ref.	< 10 ^− 4^
Yes	15.49 (10.77–22.28)	

OR: odds ratio; Q1: first quartile; Q2: second quartile; Q3: third quartile; Q4: fourth quartile; Ref.: reference.

^a^ p value was estimated using Wald’s test.

^b^ Principal component analysis (PCA) was used to identify independent dimensions of patient trust in sources of information to limit factors included in the multivariate analysis.

Analysis was performed on data weighted on age, sex and level of education of the French population.

Concerning socio-demographic factors, the respondents were more favourable to the new mandatory vaccination policy if they were men (odds ratio (OR): 1.40; 95% confidence interval (CI): 1.03–1.91), had a higher educational level (OR: 1.77; 95% CI: 1.25–2.51) and lived in an urban area (OR: 1.69; 95% CI: 1.19–2.42). Regarding sources of information on vaccination, the respondents were more favourable to the new mandatory vaccination policy if they trusted news media (OR: 2.46; 95% CI: 1.81–3.33), health professionals (OR: 18.99; 95% CI: 10.10–35.70) or institutional sources (OR: 10.63; 95% CI: 7.77–14.56). They were less in favour of the new mandatory vaccination policy if they trusted alternative health practitioners (OR: 0.51; 95% CI: 0.36–0.71) and social media networks (OR: 0.49; 95% CI: 0.24–0.98).

The numeric scale questions on vaccination’s benefits and risks were all significantly associated with an opinion on the new mandatory vaccination policy: the highest quartiles for variables concerning benefits of vaccination and the lowest quartiles concerning the probability and seriousness of side effects and the level of inconvenience were associated with a favourable opinion (Table 4).

In multivariate analysis, factors significantly associated with a favourable opinion on the new mandatory vaccination policy were: believing that vaccination brings a very important health benefit to the population (aOR: 8.17; 95% CI: 4.40–15.16), thinking that vaccines are thoroughly tested (aOR: 5.27; 95% CI: 3.54–7.85), trusting health professionals or news media regarding vaccine topics (aOR: 4.34; 95% CI: 2.26–8.32) and expecting that the most common vaccination side effects are not severe (aOR: 3.30; 95% CI: 1.91–5.72) (Table 5).

**Table 5:** Multivariate analysis for predicting favourable attitudes towards new mandatory vaccines, perception of mandatory childhood vaccination programme study, France, 2017 (n = 3,222)

Survey responses	aOR (95% CI)	p value^a^
**Sex**		
Female	NS	NS
Male		
**Level of education**		
High school diploma	NS	NS
> High school diploma		
< High school diploma		
**Place of residence**		
Rural	NS	NS
Urban		
**Feels well informed about vaccines**		
Yes	NS	NS
No		
**Trusts (grouped by principal component analysis^b^)**		
Health professionals and/or news media (Missing values: 235)	4.34 (2.26–8.32)	< 10 ^− 4^
**Perceived population health benefits**		
Q1 (least benefits)	Ref.	< 10 ^− 4^
Q2	1.53 (0.96–2.45)	
Q3	3.49 (2.18–5.59)	
Q4 (most benefits)	8.17 (4.40–15.16)	
**Perceived individual health benefit**		
Q1 (least benefits)	NS	NS
Q2		
Q3		
Q4 (most benefits)		
**Perceived level of inconvenience**		
Q1 (least inconvenient)	NS	NS
Q2		
Q3		
Q4 (most inconvenient)		
**Perceived probability of side effects**		
Q1 (least probable)	NS	NS
Q2		
Q3		
Q4 (most probable)		
**Perceived seriousness of the most common side effects**		
Q1 (least serious)	3.30 (1.91–5.72)	< 10 ^− 4^
Q2	2.46 (1.49–4.06)	
Q3	1.70 (1.04–2.80)	
Q4 (most serious)	Ref.	
**Perceived probability of serious side effect**		
Q1 (least probable)	NS	NS
Q2		
Q3		
Q4 (most probable)		
**Personal reasons for getting vaccinated**		
Obligation only	NS	NS
Protection (personal, family, population)	NS	NS
None/Don’t know	NS	NS
**Thinks vaccines are thoroughly tested**		
Yes	5.27 (3.54–7.85)	< 10 ^− 4^
No	Ref.	

NS: non-significant result (p > 0.05); OR: odds ratio; aOR: adjusted odds ratio, Q1: first quartile; Q2: second quartile; Q3: third quartile; Q4: fourth quartile; Ref.: reference.

^a^ p values were estimated using Wald’s test.

^b^ Principal component analysis (PCA) was used to identify independent dimensions of patient trust in sources of information to limit factors included in the multivariate analysis.

Analysis performed on data weighted on age, sex and level of education of the French population.

## Discussion

This work uses data from the GrippeNet.fr study to provide an overview of opinions about the new mandatory vaccination law in France, which has been in place since 1 January 2018, in the general population. In our sample, the French population was rather in favour of the extension of mandatory vaccines for children. Perception of vaccine safety and benefits were major predictors for positive opinions towards this new policy.

In our sample of the French population, the proportion in favour of vaccination was 81.7%. This global result is consistent with a random phone survey conducted in France, the French health barometer, which found that 75.1% of respondents were in favour of vaccination in general in 2016 [24]. An Italian survey, also from 2016, found that 83.7% of parents were pro-vaccination [25]. However, these positive results need to be qualified. First, not all vaccines receive a favourable opinion from the population: in 2015, another study among Grippenet.fr participants showed that only 39% of the French population have a positive opinion about influenza vaccination in France [7]. Moreover, doubts about vaccine safety remain, as demonstrated in our study, wherein a third (33.8%) of the population regarded the new vaccination policy to be a risk for children who will be vaccinated. In 2016, an international study pointed out that vaccine safety sentiment is particularly negative in France and Italy, with 41.0% and 18.7% of the population finding vaccines unsafe, respectively [1]. In addition, half of the French parents (46%) were considered vaccine hesitant, following the WHO SAGE definition in 2016 [22].

According to the SAGE working group on vaccine hesitancy, vaccination determinants belong to the ‘3Cs’ model, composed of confidence, convenience and complacency factors [4]. In our study, several variables concerning the confidence in vaccines (i.e. a perception of low severity of the most common side effects of vaccines, a belief that vaccines are thoroughly tested and confidence in health professionals and news media concerning vaccine topics) and the complacency toward vaccines (i.e. a perception that vaccination brings a very important health benefit to the population) were associated with a positive opinion of the new mandatory vaccination policy in multivariate analysis. The question regarding the convenience dimension found an association between a low level of perceived inconvenience and a favourable opinion in univariate analysis. All of these results confirmed the relevance of the ‘3Cs’ model in the field of vaccination acceptance [23]. Likewise, according to the health belief model, vaccination resulted from the balance between perceived risks and benefits [26]. Beliefs about vaccine safety and efficacy are also frequently associated with opinions on vaccination in other studies [2,27-29]. Controversies about vaccine safety are widespread on the Internet and some news media, causing doubts about vaccine safety, as demonstrated by an Italian study exploring the relationship between MMR vaccination coverage and online search trends asocial network activity on the topic ‘autism and MMR vaccine’ [5,9]. Therefore, when it comes to vaccines, reliable sources of information are crucial and delivering clear information on vaccine safety should be a priority to overcome vaccine hesitancy [3,11]. In France, Santé publique France’s *Vaccination Info Service* website was created for this purpose, but our study reveals that it remained little known by the population [12]. Further efforts are necessary to increase its diffusion and potential impact.

Health professionals play a key role in delivering information on vaccination to the population [3]; they were the most used (by 68.1% of the population) and trusted (85.4%) source of information in our study, confirming what was found previously by the 2016 health barometer in France (81.3% of parents seeking information from a physician about immunisations [24]) and by an American study (90% of parents receiving vaccine information from their child’s healthcare provider between 2002 and 2005 [30]). However, several studies conducted in France revealed a considerable level of vaccine hesitancy among general practitioners, possibly reinforcing patients’ vaccine hesitancy [31,32].

In our sample of the French population, two thirds were in favour of the new mandatory vaccines. We found a clear difference between being in favour of vaccination and being in favour of mandatory vaccination (81.7% and 64.5%, respectively), pointing to the reluctance of the population when public health interventions are of mandatory nature. More than half of the population deemed this measure authoritarian (56.9%), as opposed to allowing for individual freedom, as is frequently claimed by anti-vaccination groups. Ten years before our study, in 2008, a French opinion survey assessed that only 56.5% of the general population was in favour of mandatory vaccination. The authors suggested that this low percentage may have been the result of a fear of reduced dialogue and a lack of information shared with parents about immunisation, or perhaps that mandatory vaccination was perceived as a violation of individual rights. However, it is interesting to note that in this study another possible response to this question was to be in favour of certain specific mandatory vaccinations, but not all (35% of the study population), which is consistent with our study (28.2%). Some respondents had a negative opinion of certain vaccinations, preventing them from being in favour of the full extension of the mandatory vaccination programme [21]. In particular, HBV immunisation is frequently considered unjustified in children, because of past unfounded controversies and as the disease primarily occurs in adults [21,22]. The feeling of loss of individual choice was also described in an American study that analysed the effects and difficulties of mandatory vaccination programmes implemented in the United States (US). The authors of this study also observed a decrease in perceived necessity and an increase in safety concerns, which led to a steady increase in exemption rates in the US [17].

No socio-demographic factors were associated with a favourable opinion on mandatory vaccines’ extension in multivariate analysis. In univariate analysis we assessed that being male, having a high level of education and living in an urban area were positively associated with acceptance of mandatory vaccines’ extension. Several studies reported higher levels of confidence in vaccine safety among people with higher educational levels or income [33]. On the contrary, a recent review on determinants of parental decision-making about vaccination revealed an association between parents’ higher socio-economic status and anti-vaccination attitudes in high-income countries, such as the US, France or Italy [3]. In France, the association between high economic status and a positive opinion of vaccination was observed in 2016, and of mandatory immunisation in 2008 [21,24]. Thus, interpretation of individual determinants for predicting an opinion on vaccination remains complex and challenging [34].

In the context of political changes in vaccination policies in European countries and efforts to overcome vaccine hesitancy, this study may help to improve understandings of the dimensions that impact populations’ opinions on mandatory vaccination programmes [19]. Furthermore, this study may assist countries in deciding whether or not to implement mandatory vaccination programmes and associated measures to increase vaccination coverage.

It is important to note that we deployed our questionnaire a few months after the initial communication by the French Ministry of Health about the mandatory vaccination policy change that occurred in July 2017. This timing allowed us to gather opinions and perceptions while the change was being implemented, and was possible thanks to the use of online participatory technologies. However, the topic’s high level of coverage in the news media, concerning both the government’s commitment in favour of vaccination and the anti-vaccination movement’s claims, may have affected the population’s opinions at that time. Thus, the early timing of this study may allow it to become a reference for further studies evaluating trends in public opinion on vaccination policy.

This work is a cross-sectional, self-administered study and the global response rate of 50.5% may have induced a selection bias between respondents and non-respondents; in particular, participants might be more sensitive to health issues or more interested in the vaccination topic than non-respondents. Despite weighting our data to match the French population on age, sex and level of education, our population was still not fully representative of the French population. Influenza vaccination coverage for people ≥ 65 years was higher than in the French general population of the same age group (60.9% vs 49.7% [35]). The over-representation of vaccinated individuals in the sample is a critical point in the evaluation of the population’s opinion on vaccination policy. Adjusting for age, sex, education and vaccination status would require an age/sex/education classification of vaccinated individuals in the general population that is not yet available in France.

A bigger difference was expected between the probability of serious side effects and the probability of side effects of any type (median of 32 and 49, respectively), which suggested that respondents may have misread/misunderstood the question or that they may have found difficulty in providing an evaluation on numeric scales.

In conclusion, the French population in our sample was rather in favour of the policy to extend mandatory childhood vaccination. Perceptions seem to depend on the degree of trust in the safety and benefits of vaccination. By evaluating the general population’s opinion on mandatory vaccination, this study may contribute to guide action in order to reduce vaccine hesitancy. Long-term benefits of this measure and population acceptance should be evaluated in the near future.
